# To Assess or Not to Assess: Reconsidering Day 3 Embryo Quality in Planned Freeze-All Blastocyst Cycles

**DOI:** 10.3390/jpm14060624

**Published:** 2024-06-11

**Authors:** Alessandro Bartolacci, Daria Marzanati, Beatrice Maria Barbagallo, Lisett Solano Narduche, Giulia D’Alessandro, Stefania Esposito, Valeria Stella Vanni, Rossella Masciangelo, Davide Gentilini, Enrico Papaleo, Luca Pagliardini

**Affiliations:** 1 Obstetrics and Gynaecology Unit, IRCCS San Raffaele Scientific Institute, Via Olgettina, 60, 20132 Milan, Italy; barbagallo.beatrice@hsr.it (B.M.B.); dalessandro.giulia@hsr.it (G.D.); esposito.stefania@hsr.it (S.E.); vanni.valeria@hsr.it (V.S.V.); masciangelo.rossella@hsr.it (R.M.); papaleo.enrico@hsr.it (E.P.); pagliardini.luca@hsr.it (L.P.); 2 Department of Brain and Behavioral Sciences, University of Pavia, 27100 Pavia, Italy; daria.marzanati01@universitadipavia.it (D.M.); davide.gentilini@unipv.it (D.G.); 3 Reproductive Sciences Laboratory, Obstetrics and Gynaecology Unit, IRCCS San Raffaele Scientific Institute, Via Olgettina 60, 20132 Milan, Italy; solano.lisett@hsr.it; 4 Bioinformatics and Statistical Genomics Unit, Istituto Auxologico Italiano IRCCS, 20095 Milan, Italy

**Keywords:** embryo assessment, blastocyst, freeze-all cycles, live birth

## Abstract

Day 3 embryo quality is a predictor of in vitro fertilization (IVF) success rates in cleavage-stage embryo transfer. However, the association between day 3 embryo quality and clinical outcomes in blastocyst transfer policy is largely unknown. This retrospective study included 1074 frozen–thawed single day 5/6 blastocyst transfers between January 2019 and December 2022. Three groups were assessed depending on whether the transferred blastocyst derived from a top-quality, good-quality, or poor-quality embryo at day 3. The analysis was conducted independently for each blastocyst quality group (top, good, and poor) using multivariable logistic regression. We applied a Factorial Analysis of Mixed Data (FAMD) to reduce the potential collinearity between the covariates used in the model. All the blastocysts included in this study were obtained from the first ICSI freeze-all cycles. The cleavage and blastocysts stages were assessed between 67 ± 0.5 (day 3), 115 ± 0.5 (day 5), and 139 ± 0.5 (day 6) hours post-insemination (hpi), respectively. After adjusting for the day of transfer (day 5 or day 6) and FAMD dimensions, no statistical differences in a β-HCG, clinical pregnancy, and live birth were observed among the same-quality blastocysts derived from different day 3 embryo quality groups (top = A, good = B, and poor = C). Our findings showed that a day 3 embryo quality assessment may be unnecessary in planned freeze-all blastocyst cycles.

## 1. Introduction

The primary goal of in vitro fertilization (IVF) treatments is to achieve a healthy live birth. To increase the chances of success, transferring a single day 5 blastocyst seems to be the most effective approach. In fact, transferring blastocysts is considered more physiologically advantageous compared to transfers at the cleavage stage, as it theoretically allows for better synchronization between the embryo developmental stage and endometrial receptivity [1].

In recent years, vitrification has become the preferred method for cryopreserving blastocysts in human IVF, resulting in a high live birth (LB) rate [2,3]. Over the past decades, frozen–thawed blastocyst transfer (FBT) cycles have increased, and the success rates following FBT are comparable to, or even superior to, those of fresh embryo transfer [4,5,6,7,8]. This has led to the adoption of “freeze-all” strategies in IVF. In this approach, all embryos are electively cryopreserved, and the transfer is delayed.

Blastocyst selection is a crucial step in the IVF process. To aid in this selection process, a widely accepted morphological classification system was introduced by Gardner and Schoolcraft over a decade ago [9]. This system categorizes blastocysts based on three criteria: the degree of expansion and hatching, the quality of the inner cell mass (ICM), and the trophectoderm (TE) cells. The validity of this classification system has been supported by numerous studies and expert consensus [10,11,12,13].

Several studies showed that day 3 embryo quality could have a potential predictor of clinical outcomes in blastocyst transfer [14,15,16,17]. This could imply that incorporating both the cleavage stage and blastocyst morphology may augment the discriminatory capability of traditional blastocyst morphological assessments. On the contrary, other studies showed that performing a good-quality blastocyst transfer should be carried out regardless of the day 2/3 embryo quality [18,19,20], but data on this particular topic are still too scarce. Moreover, there is a lack of studies focusing on the predictive power of day 3 embryo assessment in poor-quality blastocyst transfer. Given the inconsistent evidence regarding this relationship, it remains uncertain whether considering the cleavage-stage morphology enhances the accuracy of the current blastocyst grading system.

In consideration of this ambiguity in the literature, to clarify the clinical value of a day 3 embryo assessment, we conducted a retrospective study involving 1074 FBTs single day 5/6 blastocyst transfers from the first ICSI freeze-all cycles. The analysis was stratified for different blastocyst quality groups (top, good, and poor quality). We tested the hypotheses that a day 3 embryo assessment has no predictive value to the blastocyst selection.

## 2. Materials and Methods

### 2.1. Study Design

This retrospective study included 1074 single day 5/6 FBTs between January 2019 and December 2022 obtained from the first ICSI freeze-all cycles. Three groups were assessed depending on whether the transferred blastocyst derived from a top-quality, good-quality, or poor-quality embryo at day 3. The analysis was conducted independently for each blastocyst quality group (top, good, and poor). The cleavage and blastocyst stages were assessed between 67 ± 0.5 (day 3), 115 ± 0.5 (day 5), and 139 ± 0.5 (day 6) hours post-insemination (hpi), respectively. The exclusion criteria were (i) ICSI cycles performed with surgically retrieved or frozen sperm, (ii) gamete donation cycles, (iii) cryopreserved–thawed oocyte cycles, and (iv) preimplantation genetic testing. No restriction criteria were adopted for maternal age. Only fully expanded vitrified blastocysts and achieving full post-warmed expansion were used in the selected cases. During the study period (3 years), no changes were introduced in the clinical and laboratory routine. New staff were introduced only after a period of training to ensure harmonization with the pre-existing operators. This study was approved by the local Institutional Review Board.

### 2.2. Ovarian Stimulation Protocol, ICSI, and Embryo Culture

All the FBTs included in this study were obtained from stimulated oocyte retrieval ICSI freeze-all cycles as previously described [21]. Either a GnRH agonist or GnRH antagonist daily protocol was used for the pituitary down-regulation. Follicular stimulation was performed with highly purified FSH (IBSA, Lodi, Italy) with a starting dose ranging from 100 to 300 IU per day, according to the hormonal and anthropometric parameters. Both the initial and adjusted doses during the treatment were chosen on a case-by-case basis according to the patients’ characteristics and response to gonadotropins. Triggering of the final oocyte maturation was performed with HP-human chorionic gonadotropin (hCG) (Gonasi; Amsa, Rome, Italy) when one or more follicles had reached a diameter of ≥17 mm. OPU was performed 35–36 h after the hCG triggering via transvaginal ultrasound-guided aspiration. ICSI was performed as previously reported [22]. Cumulus–corona–oocyte complexes were collected and washed in HTF medium with HEPES (provided by Sage In Vitro Fertilization, Inc., Trumbull, CT, USA), which was supplemented with 5 g/L of human serum albumin (HSA) (from Sage In Vitro Fertilization, Inc.). After incubating for 2–3 h in HSA-supplemented Fertilization medium (Sage In Vitro Fertilization, Inc.) under oil, the cumulus oophorus was denuded by briefly exposing it to a 40 IU/mL hyaluronidase solution (Sage In Vitro Fertilization, Inc.) in HTF medium with HEPES. The cumulus was then mechanically removed using plastic denuding pipettes on a heated-stage stereomicroscope under an IVF workstation. Following denudation, the oocytes were immediately inseminated via ICSI.

After 16–18 h, the oocytes were checked for fertilization. Normal fertilization was assessed by the presence of two pronuclei (2PN) and two polar bodies. The fertilized oocytes were cultured in micro drops of equilibrated Cleavage medium (Sage In-Vitro Fertilization, Inc., Trumbull, CT, USA) under oil. Medium change-over was performed on day 3 by transferring the embryos to a fresh culture dish prepared on day 2. All the embryos included in this study were systematically cultured to the day 5 blastocyst stage in a Blastocyst medium (Sage In-Vitro Fertilization, Inc., Trumbull, CT, USA). No changes in the embryo culture conditions were made throughout this study.

### 2.3. Embryo Morphological Assessment

The embryos were morphologically assessed on day 3 (67 ± 0.5 hpi) or day 5/6 (115 ± 0.5 and 139 ± 0.5 hpi, respectively). On day 3, the embryos were evaluated according to the number of blastomeres, the percentage of fragmentation, and the symmetry of the blastomeres. A top-quality embryo was defined as an embryo with ≥6–8 symmetrical blastomeres with no fragmentation (fragments defined as nonnuclear membrane-bound extracellular cytoplasmic structures and the fragmentation degree reflecting the percentage of the total embryo volume occupied by the fragments). A blastocyst evaluation was performed according to modified Gardner and Schoolcraft [23]. Briefly, on the morning of days 5–6 of development, the blastocysts were given a scoring based on (i) the degree of expansion and hatching status (from 1 as early to 5 as a hatched blastocyst); (ii) the inner cell mass scoring: “A” when prominent, easily discernible, with many cells that were compacted and tightly adhered together, “B” when easily discernible, with many cells that were loosely grouped, or “C” when it was difficult to discern, with few cells; and (iii) the trophectoderm scoring: “A” when many cells were forming a cohesive epithelium, “B” when few cells were forming a loose epithelium, and “C” when a small number of cells was present. According to the modified Gardner and Schoolcraft [23], an optimal blastocyst at this developmental stage is a fully expanded hatching blastocyst with an “A” inner cell mass and an “A” trophectoderm. Based on this, a “top-quality” blastocyst was defined as an expanded or hatched blastocyst with both an inner cell mass and multicellular trophectoderm scoring “A” or with only one of the two parameters scoring “B” and the other scoring “A”. In our study, the blastocysts were never frozen before the degree of expansion as 3. Overall, six embryologists performed the embryo assessment. Nevertheless, to reduce the potential bias of intra-operator variation, two senior embryologists (rigorously trained, qualified, experienced, and with more than six years of experience) graded each blastocyst simultaneously at a time.

### 2.4. Blastocyst Vitrification and Warming

Following artificial shrinkage, the expanded blastocysts were cryopreserved using a simplified vitrification protocol developed by Irvine Scientific (FUJIFILM Irvine scientific, Santa Ana, USA, https://www.irvinesci.com/, accessed on 8 April 2024). Equilibration was performed at room temperature for a duration of 10 min. The blastocysts were subsequently transferred into vitrification solution drops for 1 min before loading. Each blastocyst was cryopreserved individually. After the warming process (https://www.irvinesci.com/, accessed on 8 April 2024) using Irvine (Vit Kit-Thaw; Irvine Scientific), they were transferred into a culture dish with six 30 µL drops of blastocyst medium (Quinn’s Advantage Blastocyst Medium; CooperSurgical Fertility) under 3 mL of mineral oil (Sage, CooperSurgical Company, Fertility). All the blastocysts were cultured for at least 3 h before performing the embryo transfer.

### 2.5. Embryo Transfer and Endometrial Preparation

Single FBTs (*n* = 574 day 5 blastocyst and *n* = 500 day 6 blastocyst) were performed on day 5 or day 6 according to the guidelines of the American Society for Reproductive Medicine [24,25]. All the FBTs were performed in either a natural or an artificial cycle. In a modified natural cycle, ovulation was induced through the administration of HCG. Conversely, in artificially supplemented cycles, the endometrial preparation involved the administration of oestradiol valerate (6 mg/day) in combination with micronized vaginal progesterone (600 mg/day) and Prometrium^®^ (Meda Pharma S.p.A., Milan, Italy). Vaginal progesterone supplementation was initiated once the endometrial thickness exceeded 7 mm.

### 2.6. Outcomes Measure

The primary end-point was LB. The LB rate was defined as the number of live births out of the total number of transferred blastocysts. The secondary outcomes were positive beta human chorionic gonadotropin (β-HCG) and the clinical pregnancy (CP) rate. A positive β-HCG test was defined as a serum β-HCG concentration ≥ 100 mIU/mL, measured 14 days after the embryo transfer. Clinical pregnancy was defined as the presence of an intrauterine gestational sac with visible fetal heart activity.

### 2.7. Statistical Analysis

**Figure 1:**
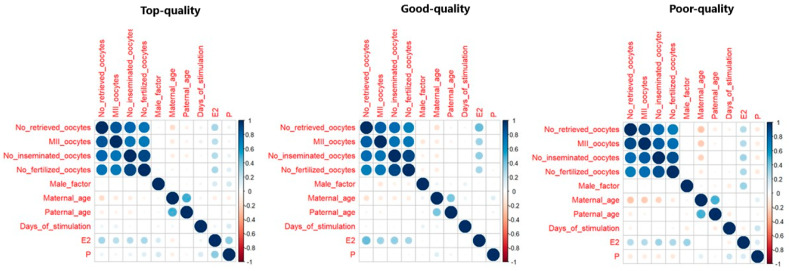
Correlogram. The correlation between the continuous variables describing the couple’s characteristics among three blastocyst quality subgroups.

A data analysis was performed with the R studio version 2022.12.0.353 (RStudio: Integrated Development for R. RStudio, PBC, Boston, MA, USA, URL http://www.rstudio.com/, accessed on 8 April 2024). All the continuous variables were expressed as means ± standard deviation (SD). The baseline characteristics of the three study groups (day 3 top quality, good quality, and poor quality) were compared with the Kruskal–Wallis non-parametric test. The cause of infertility was presented as a percentage and compared among the groups with a Chi-squared test. The predictive power of the day 3 embryo quality on β-HCG, clinical pregnancy, and live birth was evaluated by categorizing the embryos into three groups based on their quality at the blastocyst stage: top quality, good quality, and poor quality. A Generalized Linear Mixed Model (GLMM) was used to assess the association, with a binomial distribution and a logit link function. To account for multiple cycles, the couple’s identification code was included as a random effect in the model. To reduce the number of GLMM predictors and reduce collinearity within the model, a Factorial Analysis of Mixed Data (FAMD) was used (Figure 1). The variables assessed with the FAMD were the number of retrieved oocytes, inseminated oocytes, fertilized oocytes, mature (metaphase II) oocytes, total motile sperm count (million), maternal age, paternal age, days of stimulation, estrogen on the day of the ovulation trigger, progesterone concentration on the day of the ovulation trigger, and cause of infertility. All the reported *p*-values were corrected for the dimensions obtained from the FAMD, which explained more than 80% of the variance of the data, as previously described, and for the day of transfer (day 5 or day 6). A *p*-value < 0.05 was considered statistically significant. A statistical power analysis was performed using the MedCalc Software (v.19.5.3) and revealed that this study has a power equal to 90% to detect differences in live birth larger than 10% (assuming a type I error of 0.05).

## 3. Results

A total of 1074 FBTs (day 5 = 574 and day 6 = 500) were included in this study. The patient characteristics across the three groups are presented in Table 1. No significant differences were found in the maternal age at pick-up, maternal age at FBT, anti-Müllerian hormone (AMH), progesterone levels, day of stimulation, and cause of infertility. We observed a statistically significant difference in the number of oocytes retrieved (13.55 ± 6.88 group 1, 13.89 ± 6.43 group 2, and 12.76 ± 7.04 group 3; *p*-value = 0.009) and mature oocytes (10.30 ± 5.46 group 1, 10.44 ± 5.14 group 2, and 9.61 ± 5.54 group 3; *p*-value = 0.018) and the blastocyst quality among the study groups (Table 1). After adjusting for the day of transfer (day 5 or day 6) and FAMD dimensions, no statistical differences in β-HCG, CP, and LB (Table 2) were observed among the same-quality blastocysts derived from the different day 3 embryo quality groups (top = A, good = B, and poor = C). The analysis was stratified for the different blastocyst quality groups (Table 2):

(i) Top-quality blastocyst: β-HCG [day 3 quality A = reference group; day 3 quality B = OR 1.01 95% CI (0.61–1.68), *p* value = 0.957; and day 3 quality C = OR 0.74 95% CI (0.39–1.40), *p* value = 0.361], clinical pregnancy [day 3 quality A = reference group; day 3 quality B = OR 0.95 95% CI (0.58–1.57), *p* value = 0.856; and day 3 quality C = OR 0.59 95% CI (0.31–1.13), *p* value = 0.112], and live birth [day 3 quality A = reference group; day 3 quality B = OR 1.05 95% CI (0.63–1.76), *p* value = 0.856; and day 3 quality C = OR 0.66 95% CI (0.31–1.31), *p* value = 0.234].
(ii) Good-quality blastocyst: β-HCG [day 3 quality A = reference group; day 3 quality B = OR 0.73 95% CI (0.47–1.14), *p* value = 0.167; and day 3 quality C = OR 0.88 95% CI (0.55–1.40), *p* value = 0.582], clinical pregnancy [day 3 quality A = reference group; day 3 quality B = OR 0.72 95% CI (0.46–1.13), *p* value = 0.148; and day 3 quality C = OR 0.82 95% CI (0.51–1.32), *p* value = 0.414], and live birth [day 3 quality A = reference group; day 3 quality B = OR 0.78 95% CI (0.49–1.29), *p* value = 0.336; and day 3 quality C = OR 0.73 95% CI (0.43–1.25), *p* value = 0.255].
(iii) Poor-quality blastocyst: β-HCG [day 3 quality A = reference group; day 3 quality B = OR 0.93 95% CI (0.41–2.08), *p* value = 0.859; and day 3 quality C = OR 0.89 95% CI (0.41–1.96), *p* value = 0.776], clinical pregnancy [day 3 quality A = reference group; day 3 quality B = OR 1.47 95% CI (0.61–3.49), *p* value = 0.388; and day 3 quality C = OR 1.29 95% CI (0.55–3.02), *p* value = 0.559], and live birth [day 3 quality A = reference group; day 3 quality B = OR 0.71 95% CI (0.24–2.09), *p* value = 0.536; and day 3 quality C = OR 0.80 95% CI (0.28–2.27), *p* value = 0.680].

**Table 1:** Patient characteristics according to different groups.

Variables	Day 3 Top Quality	Day 3 Good Quality	Day 3 Poor Quality	*p*-Value
Frozen–thawed embryo transfer, n	348	425	301	n.a.
Maternal age at pick-up, mean ± SD	35.24 ± 3.66	35.39 ± 3.70	35.15 ± 3.92	0.698
Maternal age at FBT, mean ± SD	35.78 ± 3.71	35.99 ± 3.75	35.73 ± 3.96	0.653
AMH (ng/mL), mean ± SD	3.77 ± 3.12	4.04 ± 3.09	3.60 ± 2.76	0.241
Progesterone (ng/mL), mean ± SD	1.17 ± 1.32	1.13 ± 1.12	1.22 ± 1.01	0.477
Day of stimulation, mean ± SD	10.35 ± 3.73	10.30 ± 2.61	10.21 ± 1.67	0.949
Oocytes retrieved, mean ± SD	13.55 ± 6.88	13.89 ± 6.43	12.76 ± 7.04	0.009
Mature oocytes, mean ± SD	10.30 ± 5.46	10.44 ± 5.14	9.61 ± 5.54	0.018
Top-quality blastocyst, n (%)	120 (34.48)	148 (34.82)	67 (22.26)	0.001
Good-quality blastocyst, n (%)	179 (51.44)	205 (48.24)	150 (49.83)	0.001
Poor-quality blastocyst, n (%)	49 (14.08)	72 (16.94)	84 (27.91)	0.001
Cause of infertility				
Endometriosis, n (%)	31 (8.90)	51 (12.00)	28 (9.30)	0.358
PCO, n (%)	45 (12.93)	61 (14.35)	36 (11.96)	0.576
Tubal factor, n (%)	42 (12.06)	41 (9.65)	22 (7.31)	0.170
Poor ovarian reserve, n (%)	17 (4.88)	14 (3.29)	22 (7.31)	0.298
Male factor, n (%)	154 (44.25)	172 (40.47)	129 (42.86)	0.784
Mixed, n (%)	59 (16.98)	86 (20.24)	64 (21.26)	0.476

n.a., not applicable; n, number; SD, standard deviation; AMH, anti-Müllerian hormone; and FBT, frozen blastocyst transfer.

**Table 2:** Clinical outcomes in the studied groups.

	OR (CI 95%)	*p*-Value
Top-quality blastocyst group (n = 335)		
β-HCG		
Day 3 top quality	Ref.	
Day 3 good quality	1.01 (0.61–1.68)	0.957
Day 3 poor quality	0.74 (0.39–1.40)	0.361
Clinical pregnancy		
Day 3 top quality	Ref.	
Day 3 good quality	0.95 (0.58–1.57)	0.856
Day 3 poor quality	0.59 (0.31–1.13)	0.112
Live birth		
Day 3 top quality	Ref.	
Day 3 good quality	1.05 (0.63–1.76)	0.856
Day 3 poor quality	0.66 (0.33–1.31)	0.234
Good-quality blastocyst group (n = 534)		
β-HCG		
Day 3 top quality	Ref.	
Day 3 good quality	0.73 (0.47–1.14)	0.167
Day 3 poor quality	0.88 (0.55–1.40)	0.582
Clinical pregnancy		
Day 3 top quality	Ref.	
Day 3 good quality	0.72 (0.46–1.13)	0.148
Day 3 poor quality	0.82 (0.51–1.32)	0.414
Live birth		
Day 3 top quality	Ref.	
Day 3 good quality	0.78 (0.49–1.29)	0.336
Day 3 poor quality	0.73 (0.43–1.25)	0.255
Poor-quality blastocyst group (n = 205)		
β-HCG		
Day 3 top quality	Ref.	
Day 3 good quality	0.93 (0.41–2.08)	0.859
Day 3 poor quality	0.89 (0.41–1.96)	0.776
Clinical pregnancy		
Day 3 top quality	Ref.	
Day 3 good quality	1.47 (0.61–3.49)	0.388
Day 3 poor quality	1.29 (0.55–3.02)	0.559
Live birth		
Day 3 top quality	Ref.	
Day 3 good quality	0.71 (0.24–2.09)	0.536
Day 3 poor quality	0.80 (0.28–2.27)	0.680

SD, standard deviation; CI, confidence intervals; OR, odd ratio; FBT, frozen blastocyst transfer; and Ref., reference group.

## 4. Discussion

Currently, the discriminatory power of day 3 embryo quality on clinical outcomes in blastocyst transfer policy has not been conclusive. To contribute novel evidence in this area, we conducted a retrospective study involving 1074 day 5/6 FBTs from the first freeze-all ICSI cycles. Our study aimed to establish whether a day 3 embryo quality assessment should be considered in planned freeze-all blastocyst cycles. Several studies investigating the importance of a day 3 embryo assessment in blastocyst transfer reached contradictory results [17,18,19,26,27]. In our study, after adjusting for FAMD dimensions, no statistical differences in β-HCG, CP, and LB were observed among the same-quality blastocysts derived from different day 3 embryo quality groups (top = A, good = B, and poor = C). According to our results, one study showed no relevant impact of a day 3 embryo assessment in the same good-quality blastocyst transfer [19]. In this study, the authors included only good-quality blastocysts [19], while in our study we analyzed all three quality blastocyst groups (top, good, and poor), reaching similar results. Additionally, another study suggested the limited value of day 3 embryo assessment in predicting the live birth outcome of the single blastocyst [28]. Taken together, this evidence suggests that day 3 embryo quality may be unnecessary in blastocyst transfer policy. In contrast with our findings, two studies showed a positive correlation between the day 3 cell number and live birth in blastocyst transfer [17,26]. This discrepancy may reflect different study designs. The authors assessed two independent variables: the number of blastomeres and the percentage of fragmentation. In our study, we combined these variables into one single variable (embryo quality). According to the Istanbul Consensus [13], an embryo with 8 blastomeres with more than 25% fragmentation is considered poor quality, while one with 6/8 blastomeres without fragmentation is considered top quality. An interesting study showed that embryo morphokinetics is more important than embryo morphology in predicting live birth at the cleavage stage [29]. These findings suggest that a poor-quality embryo (eight blastomeres with more than 25% fragmentation) may have a higher live birth probability than a top-quality embryo (six and/or eight blastomeres with 0% fragmentation) [29]. This challenges the assumption that a top-quality embryo always has a superior chance of success. Our results align with this notion, revealing no significant difference between same-quality blastocysts, irrespective of whether they are derived from a top- or poor-quality embryo at day 3.

These notions are also supported by a recent time-lapse study [30]. The authors develop and validate a fully automated deep-learning model for evaluating human embryos incubated for 2, 3, and 5 days. Interestingly, the AUCs of the model based on day 3 are lower than those based on day 5, suggesting a low predictive power of a day 3 embryo assessment.

The likelihood of a top-quality embryo on day 3 reaching the blastocyst stage is superior to a poor-quality embryo [31,32], but the blastocysts obtained have the same developmental competence. One study assessing embryos with direct unequal cleavage (DUC) showed lower euploidy rates at the cleavage stage, but not at the blastocyst stage, and similar LB rates were observed if DUC blastocysts were transferred compared to non-DUC embryos [31]. Moreover, another recent study showed no negative impact on live birth and neonatal outcomes for blastocysts derived from abnormal cleavage embryos at day 3 [33].

These data suggest the theory of embryo “self-correction”. A recent review describes an evident plasticity of the human embryo, suggesting that it is detected not only as the capacity for self-correction in response to chromosomal aberrations but also as an adaptation to invasive manipulations, such as cryopreservation, ICSI, and biopsy [34]. Taken together, these data leave speculation that day 3 embryo assessments have low discriminatory power in predicting live birth in blastocyst transfer.

Our results showed lower retrieved and mature oocytes in the day 3 poor-quality group compared to the day 3 top- and good-quality groups. As a result, the percentage of top-quality blastocysts obtained was also lower in the day 3 poor-quality group. However, our data showed the same reproductive potential between blastocysts derived from poor- or top-quality day 3 embryos.

Our findings assume importance in the context of clinical practice. It is known how fluctuations in temperature, CO_2_, and O_2_ jeopardize embryo developmental competence [35,36]. In a laboratory performing static embryo assessment, not assessing embryos on day 3 could reduce the stress on the embryos by avoiding unnecessary retrievals from the incubators. Moreover, this leads to an improvement in laboratory workflow, reducing both the workload of the operator and the consumables (culture dishes). On the other hand, we are not able to provide data regarding the importance of dish renewal on day 3, but this study was not designed for that purpose.

Nevertheless, in a recent randomized control trial, a higher fertilization and embryo utilization rate was found in favor of a single-step medium compared to a sequential medium [37]. Moreover, a meta-analysis indicates that currently, there is not enough evidence to recommend the use of sequential or single-step media in extended cultures up to day 5/6, but it has been shown that there is higher blastocyst formation in single-step medium [38].

Some strengths and limitations of this study are worth commenting on. One flaw of our study is not performing an embryo assessment using time-lapse technology at the same developmental time-point, although the static observation time being very close and restricted to the Istanbul consensus guidelines may mitigate such a weakness. The retrospective nature of this study is another limitation. On the other hand, the sample size and the use of FAMD to reduce the potential collinearity between the covariates used in the model are strengths of our study.

## 5. Conclusions

In conclusion, our findings showed that a day 3 embryo quality assessment may be unnecessary in planned freeze-all blastocyst cycles. In the context of a static embryo assessment, this can reduce the stress on embryos by avoiding unwarranted retrievals from incubators. A randomized controlled trial is required to confirm our findings on the day 3 embryo assessment in a planned freeze-all blastocyst cycle.

## Data Availability

The data that support the findings of this study are available from the corresponding author, upon reasonable request.
